# Effects of Whole-Body Vibration Training on Lower Limb Blood Flow in Children with Myelomeningocele—A Randomized Trial

**DOI:** 10.3390/jcm10184273

**Published:** 2021-09-21

**Authors:** Małgorzata Domagalska-Szopa, Andrzej Szopa, Andrzej Siwiec, Ilona Kwiecień-Czerwieniec, Lutz Schreiber, Józefa Dąbek

**Affiliations:** 1 Department of Medical Rehabilitation, Medical University of Silesia in Katowice, 40-752 Katowice, Poland; mdomagalska@sum.edu.pl; 2 Department of Physiotherapy, Medical University of Silesia in Katowice, 40-752 Katowice, Poland; 3 John Paul II Pediatric Center in Sosnowiec, 41-218 Sosnowiec, Poland; sivietz@wp.pl (A.S.); i.czerwieniec@centrum-pediatrii.com.pl (I.K.-C.); 4 Department of Neurosurgery Klinikum Vest, Academic Teaching Hospital, Ruhr-University Bochum, 44801 Bochum, Germany; lutz.schreiber@klinikum-vest.de; 5 Department of Cardiology, Faculty of Health Sciences in Katowice, Medical University of Silesia, 40-635 Katowice, Poland; jdabek@sum.edu.pl

**Keywords:** spina bifida, Doppler ultrasound test, range of motion, whole-body vibration, myelomeningocele, lower limb

## Abstract

The objective of the present study was to determine the effectiveness of a three-week Whole-Body Vibration (WBV) training on the vascular blood flow of the lower limbs in children with myelomeningocele. The secondary goal was to evaluate the effect of WBV on the ROM of lower limb joints in this population. A total of 30 children with MMC (7–16 years old) were enrolled in the study. Children were randomly allocated to two groups of equal numbers, using an envelope code. The experimental group underwent a 3-week WBV training, while the control group received a 3-week conventional physiotherapy (PT) program. The examination consisted of two parts: (1) Doppler USG examination of the lower limb vascular blood flow; (2) evaluation of ROM. The results obtained revealed three main findings. First, WBV training effectively improved blood flow by increasing flow velocities in all tested arteries, while the impact of the PT program was limited to a single parameter. Second, WBV training effectively improved vascular resistance in arteries of the lower legs, while the PT program did not achieve any significant differences. Third, both types of treatment intervention significantly improved ROM in all joints of the lower limbs in MMC participants.

## 1. Introduction

Spina bifida (SB) is a congenital spinal column malformation, caused by the incomplete closure of the neural tube during embryonic development [1]. Depending on the degree of severity of the spinal cord malformation, there are three types of malformations: (1) Spina bifida occulta (SBO); (2) meningocele (MC); and (3) myelomeningocele (MMC) [2]. MMC is the most severe form of spinal cord malformation. This occurs when the spinal cord is pushed through the opening in the spine resulting in partial or complete paralysis of the parts of the body below the spinal opening. If the nerve roots are damaged or the spinal cord is dysplastic, the child will present a flaccid type of motor paralysis with a lack of sensation, which is the classic lower motor neuron presentation. If part of the spinal cord below the MMC is intact and has innervation, the result is a spastic type of motor paralysis.

Depending on the area affected by MMC, a thoracic, lumbar, or sacral spine malformation is distinguished. The higher the level of malformation, the greater the degree of neurological disorder. The most common area of involvement of MMC is the lumbosacral region. The primary clinical features evident in a child with lumbosacral MMC are disturbances of innervation of the lower limbs and spinal muscles below the spinal cord injury (paralysis and paresis); decreased or absent exteroceptive and/or proprioceptive sensation; and congenital deformities of the spine [3]. The secondary symptoms of MMC can vary widely; however, they usually include soft tissue contractures and musculoskeletal deformities of the lower limbs as a result of imbalanced muscle development, postural effects of gravity, and growth [1], as well as dysfunction of the venous muscle pump (due to muscle paralysis), and disorders of cardiovascular autonomic control.

The above disturbances and low levels of daily physical activity, accompanied by low aerobic fitness and a high body mass index (BMI) [4], can lead to a higher risk of severe vasomotor changes and peripheral blood circulation disorders in individuals with MMC. As reported by Buffart et al., cardiovascular disease (CVD) is observed more frequently among adults with MMC than in the general population [4,5].

Although there is limited information on peripheral blood circulation disorders in MMC, the study of Boot et al. confirmed the presence of poorer vascular properties in the common femoral artery (i.e., smaller diameter, lower flow, higher levels of wall shear stress) in the SB population compared to able-bodied controls as well as individuals with a spinal cord injury [6]. Our previous findings also revealed that children with MMC present poor arterial circulation in their legs [7]. Additionally, this study noted a statistically significant correlation between hip and knee contractures associated with altered blood flow velocity and resistivity of the lower limb arteries [7].

Recently, whole-body vibration (WBV) training, consisting of a rotary-type platform and a high-frequency/low-amplitude vibrator, has been proposed for physiotherapy purposes in various pediatric populations [8,9,10,11]. In the last decade, WBV training has been suggested as an effective methodology to improve clinical features in children with MMC, such as bone mineral density [9] and motor function [10,11].

These findings encouraged us to investigate the effects of WBV interventions in patients with MMC. Because it was difficult to recruit a sufficient number of participants willing to undertake vibration training as the only form of physiotherapy (PT), our previous study investigated the effectiveness of WBV training applied in conjunction with conventional PT (WBV-PT). Although our findings showed that WBV-PT significantly improved both vascular properties in individuals with MMC and the ROM of lower limb joints, the effectiveness of WBV alone has not been determined [7]. However, the application of WBV using a tilt-table proved to be so safe, easy to perform, and well tolerated by children with MMC that it convinced the participants to take part in a three-week program using only WBV training.

The objective of the present study was to determine the effectiveness of a three-week WBV program on the vascular blood flow of the lower limbs in children with MMC, using a randomized controlled trial. The secondary goal was to evaluate the effect of WBV on the range of movement (ROM) of lower limbs joints in these children.

We hypothesized that the use of WBV training can more effectively impact properties of the lower limbs’ vascular blood flow than a PT program alone, as well as that WBV might present at least the same effectiveness in improving the ROM of the lower limb joints as that of conventional PT.

## 2. Materials and Methods

### 2.1. Participants

The study design, protocol, and signing of consent forms were performed in accordance with the Code of Ethics of the World Medical Association (Declaration of Helsinki) for experiments involving humans. The procedures followed were approved by The Ethical Committee of the Medical University of Silesia (PCN/0022/KB1/64/I/19). Before the study started, the purpose, procedures, and potential benefits and risks of the study were explained to participants and their parents. Prior to participation, written consent was obtained from the parents or legal guardians of all participants.

The inclusion criteria for the current study were as follows: (1) A diagnosis of MMC; (2) age above 7 years; (3) ability to follow verbal directions; and (4) no complications beyond those common to MMC (e.g., hydrocephalus, clubfeet, Arnold–Chiari malformation). Individuals were excluded from participation if they had: (1) A history of recent surgery (at least one-year post-orthopedic surgery); (2) other acute and chronic diseases not related to MMC; (3) allergy to the gel used for Doppler ultrasound examinations; (4) lack of accessibility to the examination area of the blood vessels being studied using an ultrasound handheld device; (5) a mental disorder; or (6) a history of epilepsy.

**Figure 1:**
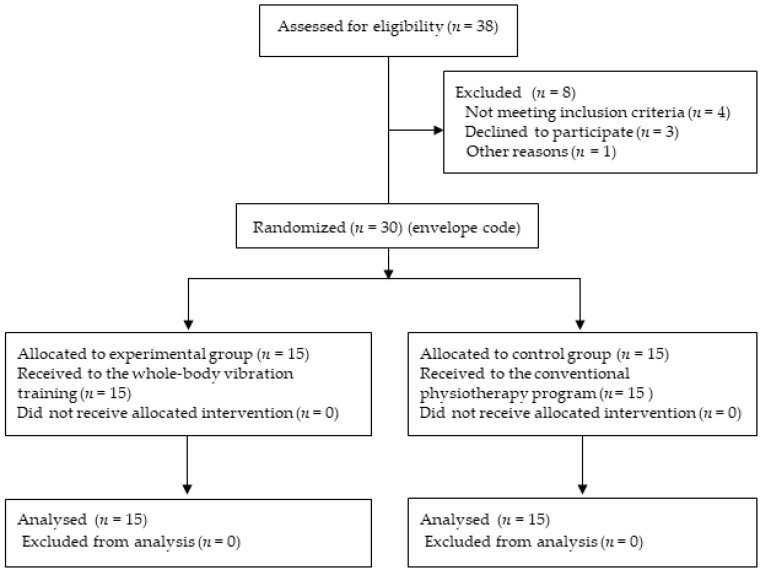
Study population.

Participants of the present study were patients from the local pediatric rehabilitation center and from special interest support groups for MMC children. Thirty-eight participants with MMC were evaluated for eligibility. Four children did not join the interventions, due to the COVID-19 pandemic and the subsequent lockdown. Three children were not evaluated at baseline and one child was not evaluated after the intervention, due to a surgical procedure. The remaining 30 children with MMC were enrolled in the study as the target population. Children were randomly allocated into two groups of equal numbers, i.e., to the intervention and control groups, using an envelope code. Envelope number 1 was assigned to the intervention group, while envelope number 2 was assigned to the control group. Nine boys and six girls, with an average age of 11.46 ± 3.50 years, were assigned to the experimental group, and ten boys and five girls, with an average age of 11.40 ± 3.74 years, were assigned to the control group. The study population is presented in Figure 1.

For each participant, the body weight, body height, and BMI were determined (Table 1). All participants were classified according to their neurological lesion level as follows: High lumbar level (HL); low lumbar level (LL); and sacral level (3). The functional level was assessed and classified according to the revised and expanded version of the Gross Motor Function Classification System in line with age (GMFCS) [12]. Participant characteristics are presented in Table 1.

### 2.2. Physical Examinations

The examination consisted of two parts: (1) Doppler ultrasound examination of the lower limb vascular blood flow (USG) and (2) evaluation of ROM. 

Both the evaluation of USG Doppler as well as the ROM examination were carried out in accordance with previously developed protocols used in our previous study [7]. 

USG Doppler examinations were performed using the ultrasound device with color flow imaging (Samsung Medison CO., LTD. Model: RS85, Seoul 05340, Korea). A linear probe with a Doppler effect was used (Linear probe 5–7, 5 MHz L743). All Doppler tests were carried out in accordance with the recommendations of the Polish Society for Vascular Surgery and the Polish Society of Phlebology, in the Triplex mode [13].

Duplex scanning was carried out separately for each lower limb and included the following arteries: (1) Superficial femoral artery (SFA; proximal section), (2) popliteal artery (POPA), and (3) anterior tibial artery (ATA, distal section) at the following points [14]:

1. SFA: Approximately 2-cm before the bifurcation of the saphenofemoral junction.
2. POPA: Central portion of popliteal fossa.
3. ATA: Near the ankle level.

The scanning site for subsequent repeated measurements was marked by a permanent marker. Based on examination of arteries, the following information was obtained: (1) Peak systolic velocity (PSV); (2) end-diastolic velocity (EDV); (3) Resistivity Index (RI); and the (4) Pulsatility Index (PI). The detailed Doppler test procedures were identical to those reported in our previous study [7]. Each examination was performed for each participant twice: At a baseline (Pre-WBV or Pre-PT) and the day after the 3-week intervention (Post-WBV or Post-PT).

An examination of the hip, knee, and ankle ROM in both legs was carried out using an accelerometer measurement system. The standardization procedures for all the ROM measurements were performed according to a previously published protocol [7]. For each test, the deficit of ROM was recorded; that is, the differences between the anatomic joint position (corresponding to an angle of 0 degrees) and the available ROM (Table 2).

### 2.3. Intervention

Participants were under a three-week treatment program at a local pediatric rehabilitation center. The participants received one of two alternative treatment programs: (1) A half-hour WBV training session 5 days per week for participants assigned envelope number 1 (intervention group) or (2) a 1-h conventional physical therapy program (PTP), 5 times weekly for participants assigned envelope number 2 (control group).

WBV was applied using a vibrating platform constructed on a tilt table (TiltTable Galileo; Novotec Medical GmbH, Pforzheim, Germany). Before the WBV training, the participant was placed in the supine position on the platform of the tilt table, which was in the horizontal position with both feet supported in parallel about 10.0-cm apart from the axis (depending on the habitual arrangement of the lower limbs). Two stabilization straps on each side provided fixation of both knees and feet to the platform. Next, the platform was slowly tilted up to the final position, which depended on the angle of the contracture of the more affected knee, so that the total sum of the angle of table tilt and the angle of knee contracture was 90°. According to the recommendations commonly used in the WBV protocol, if there was no contracture in the knees or the flexion contracture was less than 20°, the minimal knee flexion for both knees was 20°.

Because the protocol proposed to apply WBV through a vibrating platform was determined to be safe and well-tolerated by MMC patients in our previous study, we used the same protocol in the present study [7]. Before the first WBV session was performed, the patient was given a 3-min warming up session with gradually increasing frequencies of vibration (from 12 to 25 Hz) with a platform angle position of 45°. The WBV training session lasted 18 min and included 9 min of exposure to WBV and 9 min rest and consisted of the following schedule: (1) 3 min of WBV; (2) 3 min rest; (3) 3 min of WBV; (4) 3 min rest; and (5) a final 3 min of WBV and 3 min rest. The vibration platform operated at 25 Hz with a peak acceleration of 0.3 g. Each WBV training was executed by the same experienced physiotherapist, who was blinded to both physical examinations results and USG Doppler test data.

The PT program involved 1 h of personalized therapy based on isolated strengthening exercises and functional strengthening exercises to improve muscle strength and endurance in weak regions; stretching exercises to improve the active and passive ROM for the joints; exercises to correct posture while sitting and standing; exercises to improve balance and coordination; standing positioning training; and gait exercises using assistive devices such as a standing frame, parapodium, and treadmill. The PT program was performed by a team of experienced physiotherapists.

### 2.4. Statistical Analysis 

The software package SPSS v 26.0 (IBM Corp., Armonk, NY, USA) was used to carry out all statistical analyses. The sample size was calculated with G-power 3.1 software [15].

Based on the results, the sample size was calculated for the projected WBV treatment effect on the primary outcome measure, assuming an effect size of 0.5. In the main randomized controlled trial, the required number of participants was 24.

To ensure that the participants in the intervention and control groups did not differ in terms of basic characteristics, such as age, sex, height, body weight, and functional level, basic descriptive statistics were assessed along with a test of differences for a parametric variable. For categorical variables, such as sex, the presence of a neurological lesion (NLL), and functional level (GMFCS), an analysis of frequency was performed with a chi-square test of independence. In the next step, data screening among dependent variables was identified, followed by the calculation of descriptive statistics. A normality test, using the Shapiro–Wilk test, was carried out on the collected data. Descriptive statistics were computed for all data and were presented as mean (with standard deviation) or median with (range), as appropriate.

The mixed-design analysis of a variance model (Mixed Model ANOVA) was used to analyze the differences of the Doppler velocity (PSV and ED) and resistivity indices (PI and RI) for tested arteries on both lower limbs before and after the intervention, as well as the deficit of ROM of lower limb joints. There was a comparison between measurements before and after WBV (Pre-WBV and Post-WBV) for the experimental group and between measurements before and after PT (Pre-PT; Post-PT) for the control group, as well as a comparison between groups (experimental and control). For each analysis, the within-subject variable was the measurement (before and after intervention), and the between-subject variable was the group (experimental and control). The differences across the trials were considered statistically significant when the *p*-value was <0.05. 

## 3. Results

The mean age of the sample population was 11.45 (±3.56) years. There were 11 girls and 19 boys. Among participants, only 6 (20%) had normal BMI, 12 patients (40%) were overweight, and 12 patients (40%) were obese. Participants predominantly showed level II in GMFCS (60%), while the remaining 40% were evaluated as level III and IV GMFCS (26% and 14%, respectively). The most frequently involved area of neurological lesion was the lumbar (high and low) region. The demographic and anthropometric characteristics of the participants (age, sex, weight, height, BMI), functional characteristics (GMFCS), as well as neurological characteristics (NLL) are presented in Table 1. The analysis of variance showed homogeneity between experimental and control groups. The participants in both groups did not differ in terms of the subject characteristics. Detailed results of the analyses are presented in Table 1.

The results of analysis of the effect of WBV training on both Doppler velocity indices and resistivity indices showed statistically significant differences before (pre-WBV) and after (post-WBV) intervention measurements (Table 2). Both velocity indices (PSV and EDV) were statistically significantly higher in the Post-WBV assessment for all tested arteries (SFA, POPA, and ATA), and both resistivity indices (PI and RI) were statistically significantly lower for the POPA and ATA compared to the Pre-WBV assessment (Table 3). After WBV training, most parameters improved in terms of increasing both velocity indices in all tested arteries on lower limbs and reducing resistivity indices in leg arteries in the experimental group (Table 3). Generally, in the control group, there were no significant changes between the Pre-PT and Post-PT assessment of both the Doppler velocity and resistivity indices. The improvement of vascular resistance after the PT program only concerned EDV for SFA (Table 3).

**Figure 2:**
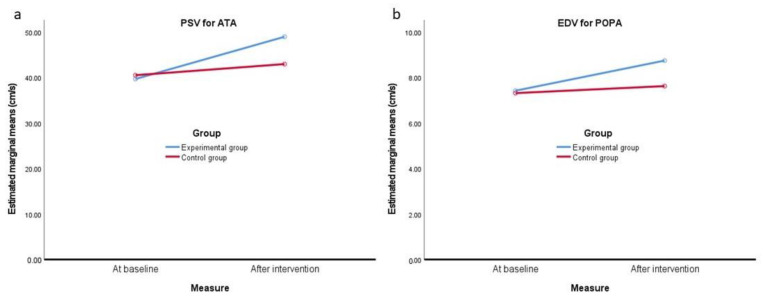
(**a**) Peak systolic velocity (PSV) for anterior tibial artery (ATA) in both lower limbs assessed at baseline and after intervention in the experimental and control groups. (**b**) End-diastolic velocity (EDV) for the popliteal artery (POPA) in both lower limbs assessed at baseline and after intervention in the experimental and control groups.

**Figure 3:**
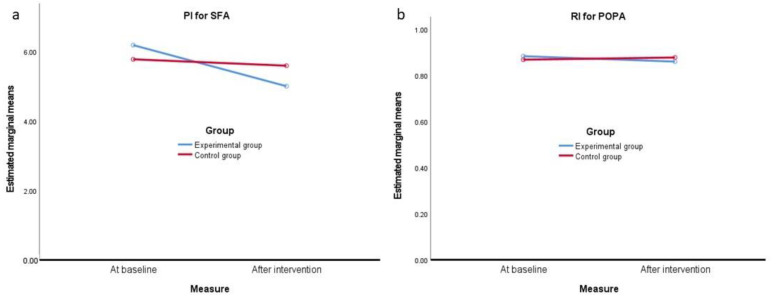
(**a**) Pulsatility Index (PI) for the superficial femoral artery (SFA) in the assessment of both lower limbs at baseline and after intervention in the experimental and control groups. (**b**) Resistivity Index (RI) for the popliteal artery (POPA) in both lower limb assessment at baseline and after intervention in the experimental and control groups.

Although there was no noticeable main effects of group, there were a few interaction effects for the combined effects of the measurement/group (Table 4), as presented in Figure 2 and Figure 3 for the differences between the assessment of the Doppler velocity indices. Examples include PSV for ATA and EDV for POPA in both lower limbs at baseline and after WBV training, which, in the experimental group, were statistically greater than in the control group (Figure 2a,b).

In the case of the analysis of Doppler vascular resistance indicators, a significant interaction effect between these factors was also observed. The differences between baseline and the final assessment of PI for SFA was significantly greater in the experimental group vs. the control group (Figure 3a), while the difference between the pre- and post-intervention RI index for POPA was significantly greater in the experimental group in comparison with non-significant differences in the control group (Figure 3b).

The effects of the intervention on deficits of ROM showed significant differences between post- and pre-intervention values of ROM in all joints of lower limbs in both the experimental and control groups (*p* < 0.05) (Table 4). Deficits in ROM for all lower limb joints after intervention (Post-WBV and Post-PT) were significantly lower (better) than those observed at baseline. However, there were no main group effects and also no effects due to interactive factors. Detailed values of the mean and standard error for ROM deficits before and after intervention are presented in Table 5.

## 4. Discussion

The purpose of this study was to assess the effectiveness of WBV training on the vascular blood flow in the lower limbs of children with MMC. The results obtained revealed three main findings. First, WBV training effectively improved blood flow by increasing flow velocities in all tested arteries, while the impact of the PT program was limited to a single parameter. Second, WBV training effectively improved vascular resistance in arteries of the lower legs, while the PT program did not achieve any significance differences. Third, both types of treatment intervention, that is, WBV training and the PT program, significantly improved ROM in all joints of the lower limbs in MMC participants. The present study documented that WBV training improved peripheral blood velocity in children with MMC by increasing both velocity indices (PSV, EDV) in all tested arteries of the lower limbs. Additionally, the obtained results revealed an improvement in the vascular resistance system after WBV training, as demonstrated by the decreasing resistivity and pulsatility indices (PI, RI) in the lower leg arteries (POPA and ATA). Although there is a lack of normative values of hemodynamic characteristics for the pediatric population, the obtained outcomes showed that most of the parameters of the lower limbs’ vascular blood, although likely far from normal, had significantly improved after WBV training.

Of note is that the conventional PT program used in the control group did not achieve similar outcomes, but the results were variable and concerned only a single parameter. This is an important finding because our previous study [7] confirmed that WBV training combined with conventional PT significantly improved vascular properties in individuals with MMC. Although the effectiveness of WBV training alone was not evaluated in the previous study, the current study clearly confirmed that WBV training is more effective in improving vascular blood velocity and the vascular resistance system of the lower limbs than the conventional PT program.

Apart from our studies, there has been no other investigation on the effects of WBV on the blood flow of the lower limbs of children with MMC; thus, it is not possible to compare our findings with other studies. Nonetheless, previous studies may have confirmed the positive influence on clinical features in individuals with MMC [8]. Specifically, these studies investigated the effects of WBV therapy on blood flow velocity and on muscular activity in adult patients with spinal cord injury and reported that WBV was an effective method to increase blood flow to the leg and could also activate the formation of muscle mass in these patients [9]. Likewise, a positive effect of WBV therapy was reported on motor function in adults with SB, which was manifested by increasing functional activity in the form of the velocity of walking. Additionally, another study [9] provided strong evidence that WBV had an influence on the bone mineral density level of children with MMC.

Although the recognition of the impact of WBV training on the vascular properties of the lower limb in individuals with MMC was the most important finding of the present study, other findings concerned the effects of WBV training on the improvement of muscle contractures in this population. Regarding the effects of using both types of therapeutic intervention on contractures of the lower limb joints, our findings clearly indicated that the ROM of all tested muscle groups after both forms of therapeutic intervention (i.e., WBV training and conventional PT) increased in comparison with those observed at baseline. Moreover, detailed data analysis indicated that, although the deficit of the knee extension in both groups was similarly reduced after the intervention (by 4 degrees on average), the flexion contractures of the knees in the experimental group at baseline were significantly larger than in controls (Table 5). It is well known that the higher the values of flexion contracture in the knees, the more difficult it is to reduce them. Thus, the influence of WBV training on the improvement of muscle contracture was clearly visible. This effect could be observed during Pre-and Post-WBV examinations. Three participants that had enrolled in the study were excluded because their flexion contractures of the knee did not permit access by an ultrasound handheld device for POPA Doppler testing. In accordance with the principles of our project, these participants received WBV training, although their data were not included in the statistical analysis. WBV training effects on knee contractures were reduced to a sufficient degree such that it was possible to perform a post-training test.

This is a very important finding, because the contractures of the lower limb joints are the main limiting functional impairment in children with MMC [10], which concerns knee flexion contractures in particular, and often involves degenerative symptoms of the bones and joints of the lower limbs and results in osteoporosis-related fractures [16,17,18]. The management of muscle and tendon contractures based on stretching is usually the main goal of any interventional program in MMC; however, it is associated with a significant risk of fragility fractures. In addition, PT requires significant physical efforts not only from MMC patients but also from physiotherapists, who often require using significant force to combat strong contractures.

The effectiveness of the PT program on the reduction of muscle contractures has already been documented in previous studies [19]. The effects of WBV training on the improvement of ROM of the lower limb joints in children with MMC were documented by our last study for the first time [7]. These findings were strongly supported by the present randomized trial. The results obtained clearly showed that the WBV intervention is at least as effective in diminishing the muscle contractures of the lower limbs in participants with MMC as conventional PT, moreover without the potential risk of fractures caused by stretching. In the context of the above reports and together with the results of the present study, the rehabilitation of MMC patients should focus not only on the management of contractures of the lower limbs and muscle activation, but also on activation of the vascular system.

Certain potential limitations of our study should be recognized. The protocol for WBV training was adapted from previously published observational studies for MMC, in which WBV was applied using a vibrating platform with a tilt table (TiltTable Galileo). The development of an optimal WBV training protocol for the improvement of lower limb blood circulation in patients with MMC needs to be extended in further studies.

Conclusions. The results of the present study provide evidence that WBV training using tilt tables significantly improved peripheral blood velocity and reduced resistivity in all tested arteries in the lower limbs of patients with MMC. Moreover, WBV training is able to improve lower-extremity muscle contractures, especially those involving the knee. Therefore, WBV training may be considered an alternative rehabilitation program for contracture management in individuals with MMC. WBV training might be a promising approach to support a comprehensive treatment program for children with SB.

## Figures and Tables

**Table 1:** Basal characteristics of participants.

Parameters		Group		Statistical Tests *p*-Values
		Group Experimental	Group Control	
Sex (N)	Boys	9	10	ꭕ^2^ = 2.17(1); 0.141
	Girls	6	5	
Age (y); mean (SD)		11.46 (3.50)	11.40 (3.74)	t = 0.50; 0.960
Height (cm); mean (SD)		143.73 (14.74)	140.93 (15.20)	t = 0.51; 0.613
Weight (kg); mean (SD)		48.90 (16.88)	48.90 (16.88)	t = 0.37; 0.715
BMI (kg/m^2^); mean (SD)		23.11 (3.73)	22.52 (4.71)	t = 0.38; 0.705
NLL; mean (Mrang)		29.73	31.27	U = 427; 0.716
GMFCS, mean (Mrang)		32.47	28.53	U = 391; 0.342

Sex (frequency) and chi-square test of independence variables. Age, height, weight, BMI (mean and standard deviation) and statistical differences for a parametric variable (t; p). Neurological characteristics lesion level (NNL) (frequency and percentage), Gross Motor Functional Classification System (GMFCS) (frequency and percentage) and statistical differences for nonparametric variable (U; p) in both groups (experimental and control.); t = Student’s *t*-test; ꭕ^2^ = chi-square test; U = Mann–Whitney test. Means (SD) of the outcome (95% CI), comparison between the groups (statistical tests and *p*-values), and functional and neurological characteristics of the children with MMC.

**Table 2:** Between repeated-measures effects (before and after intervention) and between-group effect (experimental and control group) on Doppler velocity indices (PSV and EDV) and the Doppler resistivity and pulsatility indices (RI; PI) for tested arteries on both lower limbs. Mixed Model ANOVA.

Doppler Velocity Index	Effect		df1	df2	F	*p*	η^2^
PSV (cm/s) SFA	Main	Measure	1	58	4.17	**0.046**	0.07
		Group	1	58	0.03	0.857	0.03
	Interaction	Measure × Group	1	58	1.24	0.271	0.02
EDV (cm/s) SFA	Main	Measure	1	58	15.59	**<0.001**	0.21
		Group	1	58	0.44	0.508	0.01
	Interaction	Measure × Group	1	58	0.15	0.704	0.01
PSV (cm/s) POPA	Main	Measure	1	58	3.13	**<0.001**	0.05
		Group	1	58	0.73	0.395	0.01
	Interaction	Measure × Group	1	58	0.73	0.397	0.01
EDV (cm/s) POPA	Main	Measure	1	58	13.99	**<0.001**	0.20
		Group	1	58	0.79	0.377	0.01
	Interaction	Measure × Group	1	58	5.43	**0.023**	0.09
PSV (cm/s) ATA	Main	Measure	1	58	9.40	**0.003**	0.14
		Group	1	58	1.71	0.196	0.03
	Interaction	Measure × Group	1	58	3.19	**0.049**	0.06
EDV (cm/s) ATA	Main	Measure	1	58	11.70	**0.001**	0.17
		Group	1	58	1.14	0.291	0.02
	Interaction	Measure × Group	1	58	0.59	0.446	0.01
PI SFA	Main	Measure	1	58	7.33	**0.009**	0.11
		Group	1	58	0.05	0.818	0.01
	Interaction	Measure × Group	1	58	3.91	**0.050**	0.06
RI SFA	Main	Measure	1	57	32.31	**<0.001**	0.36
		Group	1	57	0.02	0.900	<0.01
	Interaction	Measure × Group	1	57	0.74	0.395	0.01
PI POPA	Main	Measure	1	57	5.13	**0.027**	0.08
		Group	1	57	0.29	0.592	<0.01
	Interaction	Measure × Group	1	57	0.38	0.537	0.01
RI POPA	Main	Measure	1	58	2.12	0.151	0.03
		Group	1	58	0.06	0.811	<0.01
	Interaction	Measure × Group	1	58	11.54	**0.001**	0.16
PI ATA	Main	Measure	1	56	4.22	**0.045**	0.07
		Group	1	56	0.79	0.377	0.01
	Interaction	Measure × Group	1	56	0.34	0.560	0.01

Superficial femoral artery (SFA); popliteal artery (POPA); anterior tibial artery (ATA); peak systolic velocity (PSV); end˗diastolic velocity (EDV); resistivity index (RI); pulsatility index (PI). The statistically significant differences are printed in bold; results for RI ATA were not reported due to a breach of the assumption of homogeneity of variance between groups.

**Table 3:** Between repeated-measures effects (before and after intervention) and between-group effects (experimental and control groups) on Doppler velocity indices (PSV and EDV) and the Doppler resistivity and pulsatility indices (RI; PI) for tested arteries on both lower limbs before (Pre-WBV) and after Whole Body Vibration training (Post-WBV); and before (Pre-PT) and after physiotherapy program (Post-PT). Mixed Model ANOVA.

Parameters	Experimental Group				Control Group			
	Pre-WBV (*n* = 30)		Post-WBV (*n* = 30)		Pre-PT (*n* = 30)		Post-PT (*n* = 30)	
	M	SE	M	SE	M	SE	M	SE
Doppler velocity index								
PSV SFA	70.55	2.98	**75.30** ^a^	3.09	71.49	2.98	72.90	3.09
ED SFA	7.53	0.61	**9.97** ^a^	0.61	7.31	0.61	**9.32** ^b^	0.61
PSV POPA	59.98	2.86	**66.50** ^a^	3.20	59.10	2.86	61.38	3.20
ED POPA	7.42	0.51	**8.74** ^a^	0.53	7.31	0.49	7.62	0.52
PSV ATA	39.65	1.68	**48.93** ^a^	2.17	40.49	1.68	42.93	2.17
ED ATA	4.41	0.34	**6.31** ^**a**^	0.52	4.30	0.34	5.50	0.52
Doppler resistivity index								
PI SFA	6.17	0.35	4.99	0.31	5.76	0.35	5.58	0.31
RI SFA	0.91	0.01	0.86	0.01	0.90	0.01	0.87	0.01
PI POPA	9.04	0.86	**6.97** ^a^	0.66	9.04	0.88	7.86	0.67
RI POPA	0.88	0.01	**0.86** ^a^	0.01	0.87	0.01	0.88	0.01
PI ATA	9.86	0.96	**7.43** ^**a**^	0.81	9.68	0.96	8.72	0.81

M—mean; SE—standard error; Superficial femoral artery (SFA); popliteal artery (POPA); anterior tibial artery (ATA); peak systolic velocity (PSV); end˗diastolic velocity (EDV); resistivity index (RI); pulsatility index (PI); The statistically significant differences are printed in bold; ^a^—difference between Pre-WBV and Post-WBV; ^b^—difference between Pre-PT and Post-PT. Bonferroni post hoc test. Results for RI_ATA were not reported due to breach of the assumption of homogeneity of variance between groups.

**Table 4:** Between repeated-measures effects (before and after intervention) and between-group effects (experimental and control group) on deficits of the ROM of the joints on both lower limbs. Mixed-Model ANOVA.

Model	Effect		df1	df2	F	*p*	η^2^
Hip flexion (°)	Main	Measure	1	58	52.27	**<0.001**	0.47
		Group	1	58	1.38	0.245	0.02
	Interaction	Measure × Group	1	58	1.42	0.237	0.02
Hip extension (°)	Main	Measure	1	58	35.84	**<0.001**	0.39
		Group	1	58	0.12	0.727	0.002
	Interaction	Measure × Group	1	58	0.11	0.741	0.002
Knee flexion (°)	Main	Measure	1	58	36.47	**<0.001**	0.39
		Group	1	58	1.92	0.171	0.03
	Interaction	Measure × Group	1	58	1.12	0.294	0.02
Knee extension (°)	Main	Measure	1	58	115.01	**<0.001**	0.66
		Group	1	58	2.37	0.129	0.04
	Interaction	Measure × Group	1	58	0.21	0.845	0.001
Ankle Dorsiflexion (°)	Main	Measure	1	58	14.66	**<0.001**	0.20
		Group	1	58	0.163	0.688	0.003
	Interaction	Measure × Group	1	58	0.19	0.660	0.003
Ankle Plantarflexion (°)	Main	Measure	1	58	28.11	**<0.001**	0.33
		Group	1	58	0.97	0.327	0.02
	Interaction	Measure × Group	1	58	0.74	0.393	0.01

(°)—degree; The statistically significant differences are printed in bold.

**Table 5:** Deficit of the ROM of lower limbs (mean and standard error) and variables compared before (Pre˗WBV) and after Whole-Body Vibration Training (Post-WBV) in the experimental group and before (Pre-PT) and after physiotherapy program (Post˗PT) in the control group.

Parameters	Group Experimental				Group Control			
	Pre-WBV (*n* = 30)		Post-WBV (*n* = 30)		Pre-PT (*n* = 30)		Post-PT (*n* = 30)	
	M	SE	M	SE	M	SE	M	SE
Hip flexion (°)	11.27	1.66	**9.67** ^a^	1.51	8.97	1.66	**6.73** ^**b**^	1.50
Hip extension (°)	32.73	1.57	**31.50** ^a^	1.52	31.90	1.60	**30.79** ^**b**^	1.55
Knee flexion (°)	9.57	1.52	**7.66** ^a^	1.27	6.57	1.52	**5.23** ^**b**^	1.26
Knee extension (°)	21.53	2.07	**17.06** ^a^	2.00	17.23	2.06	**12.60** ^**b**^	2.00
Ankle Dorsiflexion (°)	10.13	2.73	**9.37** ^a^	2.53	11.73	2.73	**10.77** ^**b**^	2.53
Ankle Plantarflexion (°)	30.20	4.34	**28.77** ^**a**^	4.21	24.03	4.34	**23.00** ^**b**^	4.21

M—mean; SE—standard error; (°)—degree; The statistically significant differences are printed in bold; ^a^ difference between Pre-WBV and Post-WBV; ^b^ difference between Pre-PT and Post-PT. Bonferroni post hoc test.

## Data Availability

The data supporting the results of this study are available from the corresponding author upon reasonable request from any qualified investigator.

## References

[1] Sandler A.D. (2010). Children with spina bifida: Key clinical issues. Pediatr. Clin. N. Am..

[2] Rossi A., Biancheri R., Cama A., Piatelli G., Ravegnani M., Tortori-Donati P. (2004). Imaging in spine and spinal cord malformations. Eur. J. Radiol..

[3] Vinck A., Nijhuis-van der Sanden M.W., Roeleveld N.J., Mullaart R.A., Rotteveel J.J., Maassen B.A. (2010). Motor profile and cognitive functioning in children with spina bifida. Eur. J. Paed. Neurol..

[4] Buffart L.M., van den Berg-Emons R.J., Burdorf A., Janssen W.G., Stam H.J., Roebroeck M.E. (2008). Cardiovascular disease risk factors and the relationships with physical activity, aerobic fitness, and body fat in adolescents and young adults with myelomeningocele. Arch. Phys. Med. Rehabil..

[5] Buffart L.M., Roebroeck M.E., Rol M., Stam H.J., van den Berg-Emons R.J. (2008). Triad of physical activity, aerobic fitness and obesity in adolescents and young adults with myelomeningocele. J. Rehabil. Med..

[6] Boot C.R.L., van Langen H., Hopman M.T.E. (2003). Arterial vascular properties in individuals with spina bifida. Spinal Cord.

[7] Szopa A., Domagalska-Szopa M., Siwiec A., Kwiecień-Czerwieniec I. (2021). Effects of whole-body vibration-assisted training on lower limb blood flow in children with myelomeningocele. Front. Bioeng Biotechnol..

[8] Herrero A.J., Menendez H., Gil L., Martin J., Martin T., Garcia-Lopez D., Gil-Agudo A., Marin P.J. (2011). Effects of whole-body vibration on blood flow and neuromuscular activity in spinal cord injury. Spinal Cord.

[9] Emara H.A. (2014). Effect of whole body vibration on bone mineral density in children with myelomeningocele. Dent. Med Sci..

[10] Stark C., Hoyer-Kuhn H.K., Semler O., Hoebing L., Duran I., Cremer R., Schoenau E. (2015). Neuromuscular training based on whole body vibration in children with spina bifida: A retrospective analysis of a new physiotherapy treatment program. Childs Nerv. Syst..

[11] Saavedra S.L., Teulier C., Smith B.A., Kim B., Beutler B.D., Martin B.J., Ulrich B.D. (2012). Vibration-induced motor responses of infants with and without myelomeningocele. Phys. Ther..

[12] Palisano R.J., Rosenbaum P., Bartlett D., Livingston M.H. (2008). Content validity of the expanded and revised Gross Motor Function Classification System. Dev. Med. Child. Neurol..

[13] Małek G., Elwertowski M., Nowicki A. (2014). Standards of the Polish Ultrasound Society—Update. Ultrasound examination of the aorta and arteries of the lower extremities. J. Ultrason..

[14] Hwang J.Y. (2017). Doppler ultrasonography of the lower extremity arteries: Anatomy and scanning guidelines. Ultrasonography.

[15] Cunningham J.B., McCrum-Gardner E. (2007). Power, effect and sample size using GPower: Practical issues for researchers and members of research ethics committees. Evid. Based Midwifery.

[16] Apkon S.D., Fenton L., Coll J.R. (2008). Bone density in children with myelomeningocele. Dev. Med. Child. Neurol..

[17] Okurowska-Zawada B., Konstantynowicz J., Kułak W., Kaczmarski M., Piotrowska-Jastrzębska J., Sienkiewicz D., Paszko-Patej G. (2009). Assessment of risks factors for osteoporosis and fractures in children with meningomyelocele. Adv. Med. Sci..

[18] Pauly M., Cremer R. (2013). Levels of mobility in children and adolescents with spina bifida-clinical parameters predicting mobility and maintenance of these skills. Eur. J. Pediatr. Surg..

[19] Oliveira A., Jácome C., Marques A. (2014). Physical fitness and exercise training on individuals with spina bifida: A systematic review. Res. Dev. Disabil..

